# *Mycobacterium liflandii* Infection in European Colony of *Silurana tropicalis*

**DOI:** 10.3201/eid1305.060625

**Published:** 2007-05

**Authors:** Patrick Suykerbuyk, Kris Vleminckx, Frank Pasmans, Pieter Stragier, Anthony Ablordey, Hong Thi Tran, Katleen Hermans, Michelle Fleetwood, Wayne M. Meyers, Françoise Portaels

**Affiliations:** * Institute of Tropical Medicine, Antwerp, Belgium; † Flanders Interuniversity Institute for Biotechnology of Ghent University, Zwijnaarde, Belgium; ‡ Ghent University, Merelbeke, Belgium; § Noguchi Memorial Institute for Medical Research, Legon, Ghana; ¶ Armed Forces Institute of Pathology, Washington, DC, USA

**Keywords:** Mycobacterium liflandii, Silurana tropicalis, epizootic, mycobacteriosis, dispatch

## Abstract

*Mycobacterium liflandii* causes a fatal frog disease in captive anurans. Here we report, to our knowledge, the first epizootic of mycobacteriosis in a European colony of clawed frogs (*Silurana tropicalis*), previously imported from a United States biologic supply company. Our findings suggest the emerging potential of this infection through international trade.

Many species of nontuberculous mycobacteria inhabit the environment. *Mycobacterium fortuitum*, *M. chelonae, M. marinum,* and *M. xenopi* are some of the mycobacteria that infect amphibians, causing subcutaneous nodules, edema, and chronic wasting (*1*).

The aquatic, pipid frog, *Silurana tropicalis*, is an emerging laboratory model for genetic and embryologic/ontogenetic research. Although smaller than the related *Xenopus laevis*, *S. tropicalis* has research advantages: diploidy and brief maturation time make this species ideal for genetic analyses over multiple generations (*2*).

In 2004, Trott et al. characterized a new mycobacterial pathogen in pipid frog colonies (*3*), now named *M. liflandii* (*4*). On Middlebrook 7H11 agar supplemented with oleic acid, albumin, dextrose, and catalase, *M. liflandii* form rough, nonpigmented, slightly buff-colored colonies. Visible colonies develop after 30 to 35 days at 28°C on Löwenstein-Jensen (LJ) medium (*3*). This *M. ulcerans*–like mycobacterium produces a plasmid-encoded toxin, mycolactone E, which is less cytopathogenic than mycolactone A/B, produced by African *M. ulcerans* (*4*). *M. liflandii* infection in frogs manifests as cutaneous lesions, coelomitis, and bloating, with a high death rate (*3*).

We investigated an epizootic of *M. liflandii* in a colony of African tropical clawed frogs (*S. tropicalis*) in a European research laboratory. With the rising popularity of this vertebrate laboratory model and the foreseen establishment of stock centers for mutant or transgenic animals, the epizootiology of this emerging disease must be defined so that preventive measures may be instituted.

## The Study

In November 2004, we began to study an epizootic mycobacteriosis in a colony of imported captive *S. tropicalis,* the African tropical clawed frog. The Department of Molecular Biomedical Research, Flanders Interuniversity Institute for Biotechnology of Ghent University, Belgium, had imported *S. tropicalis* frogs from a supplier in the United States in September 2004. Within 5 weeks, some animals became lethargic with signs similar to those described by Trott et al.: loss of diving reflex, bloating, and ulcerative skin lesions (*3*). An average number of 2 deaths each week were reported in a colony of 300 specimens. Preliminary examination of 2 affected animals did not show chytridiomycosis; iridoviral infection; common bacterial infections of liver, lungs and kidneys; chlamydophila infection; or intestinal parasites.

From November 2004 through April 2005, 19 visually affected and visually unaffected specimens of *S. tropicalis,* 2 tadpoles, and 4 tank water samples were selected for detailed examination for mycobacteria.

**Figure 1:**
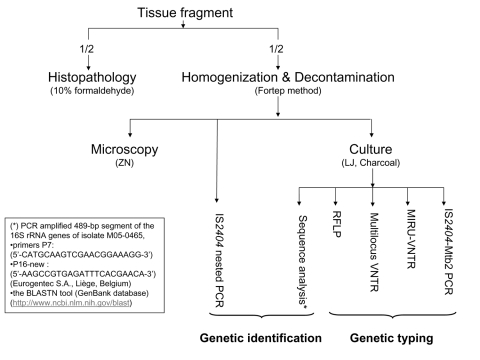
Flowchart of performed tests. ZN, Ziehl-Neelsen staining; LJ, Löwenstein-Jensen medium, charcoal medium, LJ; Middlebrook 7H11 acidified with phosphoric acid, supplemented with sheep blood and charcoal; RFLP, restriction fragment length polymorphism; VNTR, multilocus variable-number of tandem repeats; MIRU, mycobacterial interspersed repetitive unit (MIRU)-VNTR.

The frogs were euthanized and dissected, and selected organs and fluid were removed aseptically (liver, lungs, gallbladder, gastrointestinal tract, spleen, kidneys, fat body, ovary, oviduct, tibia, and coelomic fluid). Each of the specimens was divided into 2 equal parts, half for histopathologic analysis, and half for preparation of decontaminated suspensions for culture and microscopic examination (*3*,*5*–*7*). Water samples were concentrated by filtration as described by Iivanainen et al. (*7*), and suspensions were made from the complete tadpoles (*6*). Further analyses were performed as described for the decontaminated frog suspensions. DNA for genetic analyses was extracted from the suspensions and pure cultures as described previously (*6*,*8*). *M. liflandii* was identified by IS*2404* nested PCR and sequence analysis of 16S rRNA gene (*9*,*10*). A combination of 4 genetic typing assays (including 3 previously investigated in *M. ulcerans)* was used to type *M. liflandii* (*3*,*9*,*11*,*12*). A flowchart of the performed tests is shown in Figure 1.

All visually affected specimens showed positive results for at least 1 organ and for at least 2 of the following tests: microscopy (Ziehl-Neelsen staining), in vitro cultivation (LJ medium and charcoal medium), IS*2404* nested PCR, and histopathologic examination. Noteworthy, all ovarian tissue of the 11 visually affected specimens showed positive results for at least 2 tests. Three of 8 visually unaffected specimens showed positive results for at least 1 organ (including the ovary) and for 1 test. All tadpoles and water samples showed negative results for all tests.

**Figure 2:**
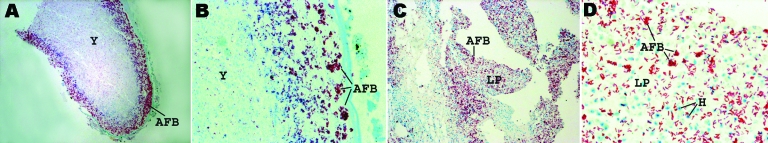
A) Oviduct, focally expanded by collections of macrophages and yolk (Y) material. Note acid-fast bacilli (AFB) throughout specimens, but concentrated at the periphery (Ziehl-Neelsen [ZN] stain ×25). B) High-power magnification of periphery of oviduct containing macrophages and yolk (Y) material; AFB are concentrated at the periphery (ZN stain ×300). C) Gallbladder with papillary hyperplasia of the mucosa. Note masses of AFB in the lamina propria (LP) of the mucosa (ZN stain ×50). D) High-power magnification of the lamina propria of the gallbladder mucosa showing large numbers of AFB and heterophils (H) (ZN stain ×300).

Histopathologic evaluation showed many acid-fast bacilli (AFB) in the oviduct lumen (Figure 2). Numerous AFB were found in the kidney tubules, on the surface epithelium, and in the lumens of the gallbladder, stomach, intestine, and oviduct. Papillary hyperplasia of the gallbladder mucosa was marked, and the lamina propria was expanded by heterophils and many AFB (Figure 2). Lung parenchyma, liver, femur, and tibia were normal and free of AFB.

We identified the causative pathogen as *M. liflandii* in all frogs: by growth on charcoal medium, by restriction fragment length polymorphism, and by sequence analysis. Isolate M05–0456 had a similarity value of 100% with *M. liflandii* (GenBank accession no. AY845224.1). Growth on charcoal medium can be considered as an additional identification criterion for *M. liflandii* because growth on charcoal is better than on LJ medium (*3*), differentiating *M. liflandii* from *M. ulcerans*. Clinical isolates of *M. ulcerans* are grown readily on LJ medium but never on charcoal medium. The antibiogram of strain M04–2878 showed resistance to isoniazid, ethambutol, rifampin, clarithromycin, and ethionamide. In each of the genotyping assays, *M. liflandii* produced profiles that were distinct from those of *M. ulcerans* (data not shown). None of the laboratory staff who handled the anurans exhibited any signs of a mycobacterial disease.

## Conclusions

The first epizootic of *M. liflandii* infection was reported by Trott et al. in 2004 in pipid frog colonies in the United States (*3*). To our knowledge, our report is the first account of *M. liflandii* disease in a colony of captive *S. tropicalis* frogs in Europe. We do not know the prevalence of *M. liflandii* infection in the colony, but we believe it was very high because 3 of 8 clinically healthy frogs were positive for *M. liflandii* by at least 1 test.

The genetic and phenotypic identification of *M. liflandii* as causative agent of the epizootic, the fact that cases of *M. liflandii* infection have not been reported in Europe to date, the strikingly similar signs and disease progress (*3*), and the probability that the frogs were imported from the same supplier (*3*) all suggest that some members of the imported *S. tropicalis* colony were infected with *M. liflandii* before arrival in Europe. Crowding and stress associated with captivity may have contributed to spread of infection within the colony. How and where the imported frogs became infected remains unknown (*3*). Additionally, during an extensive study in the Democratic Republic of Congo, Portaels (*13*) isolated 956 mycobacterial strains from the environment from Buruli ulcer–endemic regions. Among the unknown species, none was characterized as a *M. ulcerans*–like mycobacterium. To our knowledge, no *M. liflandii* infection, in humans or wild anurans, has been reported from Africa. We have confirmed that all isolates from Buruli ulcer patients and environmental samples analyzed by our laboratory were true *M. ulcerans* infections and not IS*2404* PCR-positive *M. ulcerans*–like mycobacteria (unpub. data).

The apparently enzootic character of *M. liflandii* infection in different *S. tropicalis* breeding companies in the United States (*3*,*4*), and the exchange of transgenic or mutant *S. tropicalis* lines between research laboratories, may pose a serious threat for the international research community working with this emerging laboratory model. Difficulties in detecting the pathogen in visually unaffected specimens and the high infection rate call for urgent efforts in the management of this epizootic disease. Thus far, no preventive measures or treatment for this amphibian mycobacteriosis are known (*3*,*4*,*14*). Resistance to antimycobacterial agents by environmental mycobacteria is not unusual and has been reported previously (*15*).

We propose examining the oocytes of newly imported frogs as an intervening noninvasive screening method on a regular basis, noting that all affected frogs reported in both intercontinental epizootics were females (*3*), oocytes from living adult *S. tropicalis* are easily obtained for research purposes (*2*), and ovarian tissue was positive for all visually affected specimens and for 1 of 3 positive visually unaffected specimens. However, further studies are needed to determine the role of oocytes in the epizootics of this emerging frog disease, especially in the evaluation of our proposed screening method. To prevent the infection of existing stocks with wild-caught frogs of unknown origin, we further recommend the importation of only certified pathogen-free laboratory-bred specimens from recognized biologic suppliers. Recently, Tarigo et al. reported a frog mycobacteriosis in an adult female, albino South African clawed frog (*X. laevis*) in a research colony at North Carolina State University (*14*). The etiologic agent was identified as *M. marinum* complex on the basis of mycobacterial culture, but genetic analyses were not performed to exclude *M. liflandii* infection. To avoid further spread of this disease, every new outbreak of *M. liflandii* infection in pipid frogs or other anuran species should be reported to relevant authorities and research communities. Until more is known about this epizootic and its prevention and treatment, caution must be exercised in transportation, husbandry, and human contact with these animals (zoonotic potential). We do not know at this stage whether the importation of frogs contaminated by *M. liflandii* represents a danger for wild or autochthonous frogs. Further investigation is required to establish this.
